# Gait Speed Reserve in the general population-based ‘Good Aging in Skåne’ cohort study—distribution and associated factors

**DOI:** 10.1007/s11357-024-01318-6

**Published:** 2024-08-27

**Authors:** Beata Lindholm, Rani Basna, Henrik Ekström, Sölve Elmståhl, Arkadiusz Siennicki-Lantz

**Affiliations:** 1 Department of Clinical Sciences Malmö, Cognitive Disorders Unit, Lund University, 214 28 Malmö, Sweden; 2 Department of Neurology, Rehabilitation Medicine, Memory Disorders and Geriatrics, Skåne University Hospital Malmö, 205 02 Malmö, Sweden; 3 Division of Geriatric Medicine, Department of Clinical Sciences in Malmö, Lund University, 214 28 Malmö, Sweden

**Keywords:** Gait Speed Reserve, Physiological reserve, Aging; Frailty, Mixed model

## Abstract

Gait Speed Reserve (GSR) expresses a difference between fast and comfortable gait speed and may have an impact on everyday functioning. It was also hypothesized as a useful proxy measure of physiological reserve. However, height-normalizing values of GSR and its associated factors have not been evaluated in a general population of older adults. Therefore, we aimed to investigate the distribution of height-normalized GSR (HN-GSR) in an elderly population-based cohort from urban and rural areas (*n* = 4342) aged 60–93 years and evaluate associated physiological and lifestyle factors. Using linear mixed models, we identified gender and nine modifiable factors as significantly associated with HN-GSR across four age groups. Better handgrip strength, cognition and standing balance, higher physical activity level, larger calf circumference, and less smoking had positive associations with HN-GSR, while female gender, more leg pain, higher weight and, alcohol consumption had opposite effects. The Marginal R2 imply that this model explained 26% of the variance in HN-GSR. Physical activity and handgrip strength varied across age groups in impact on HN-GSR. The differences were however comparatively minor. In this large cohort study of older adults, we proposed for the first time that factors associated with HN-GSR represented multi-domain features that are in line with previous findings reported for GSR. Measuring HN-GSR/GSR may help clinicians identify early physiological impairments or unhealthy lifestyle habits, especially among older women, and may also have safety implications in daily life. Further work is needed to find out if measuring HN-GSR/GSR may be useful in identifying adverse health outcomes and overall physiological reserve.

**Supplementary Information:**

The online version contains supplementary material available at 10.1007/s11357-024-01318-6.

## Introduction

Normal walking function is fundamental for a physically independent lifestyle and a predictor of overall health status [1]. Walking speed is the result of a complex interplay of multiple body structures and functions and usually becomes more demanding with older age [2–5]. In clinical settings, comfortable gait speed (CGS), also called “normal”, “usual”, “preferred” or “self-selected”, is frequently used as an indicator for changes in gait and balance function. The inability to walk fast enough may impact everyday functioning and safety. For example, a gait speed of 1.2 m/s is required to cross a street in the time allotted at signalized intersections [6, 7]. Therefore, one’s CGS and ability to speed up and walk fast are of particular interest. Consequently, fast gait speed (FGS) also called “maximal” is also frequently monitored in addition to CGS. It is well-known that CGS and FGS are strongly correlated [8, 9]. When evaluating the significance of CGS for FGS in a healthy older population, based on the reported linear regression coefficient, FGS could be described as 1.07 times the CGS [10]. The presented model, adjusted for gender and age, explained only 60% of the variance in FGS suggesting that the contribution of additional factors is possible.

Another approach for the evaluation of the relationship between CGS and FGS is to calculate their raw (*actual*) difference, the so-called Gait Speed Reserve (GSR) [9, 11–13]. The ability to increase gait speed requires a general effort across many body systems and, besides the impact on everyday functioning may be a useful proxy measure of maximal capacity to function beyond the basal level (so-called physiological reserve) [14–16]. GSR was suggested as a better indicator of early stages of frailty than CGS in older women [12]. However, GSR and its associated factors are still poorly evaluated. Davis et al. [9] examined a large, older population and proposed better handgrip and lower body strength as well as greater height as significantly associated factors with increased GSR, CGS, and FGS. Seeing GSR being less predictable than CGS and FGS authors suggested including of additional modifiable factors in the models. Measuring calf circumstance in addition to handgrip strength and gait speed is recommended for assessing the severity of sarcopenia — a well-known musculoskeletal disease generally defined by the age-related progressive loss of muscle mass and strength [17]. Early cognitive impairment is closely related to a decrease in gait speed [18]. Chronic pain, an underdiagnosed age-related burden [19] may impact both motor and cognitive function [17]. Also, one’s lifestyle has an impact on general physical status and walking function. Physical activity level, weight, smoking habits, and alcohol consumption were suggested as four important lifestyle factors in this context [20].

Finally, due to the significance of height for GSR, David’s et al. suggested using *height-normalized* gait speeds in analysis [9]. The use of *height-normalized* gait speed values in clinical settings was also recommended by some authors before [4, 5, 9, 21]; however, neither *height-normalized* GSR nor its associated factors were evaluated until now. Based on this background, we aimed to investigate the distribution of *height-normalized* GSR in a large population-based cohort aged 60–93 years and evaluate associated physiological and lifestyle factors.

## Methods

### Study population

The GÅS study is a longitudinal public health study, a part of the Swedish National Study on Aging and Care [22]. GÅS includes randomly selected subjects from the Swedish National Municipality Register of four rural and one large urban general population of the southern Swedish province of Skåne. Subjects at age 60–93 years were invited by letter and those who did not respond initially were invited again by letter and telephone. Invitees were considered non-eligible if they were deceased, had moved or were otherwise unreachable within 90 days of invitation. Participants who confirmed the participation in the study (*n* = 5804) were examined at three separate surveys and included 2931 subjects in wave I (response rate 60%; subgroups at age (± 1 yr): 60, 66, 72, 78, 81, 84, 87, 90, and 93 years old; examination start in 2001), 1523 subjects in wave II (response rate 60%; subgroups at age: 60, 66 and 81 years; examination start in 2006) and 1350 subjects in wave III (response rate 70%; subgroups at age: 60 and 81 years; examination start in 2012). Each wave took approximately 3.5 years to complete. Of a total 5804 examined men and women, 1462 were excluded from the analysis due to missing data on the dependent variable (*height-normalized* GSR, hereafter called HN-GSR). Finally, 4342 were included in the study (wave I: 2325, wave II: 976 and wave III: 1041).

### Assessments

All the assessments were made according to a predefined research protocol scheme and took place either at the research outpatient clinic or in the individual’s home to encourage participation of frail adults. All subjects underwent an examination performed by a trained test administrator and a registered nurse following the same study protocol during all surveys. The self-administered part of the assessment was comprised of questionnaires consisting of demographic data, pain [22] physical activity level and life style (alcohol and tobacco consumption) [23]. A registered nurse performed examination that included measurement of cognition [24] CGS and FGS [25], standing balance [26], handgrip strength [27] and anthropometric information, such as calf circumference, weight and height [28]. Further details are presented in Table 1.

**Table 1:** Included assessments

Assessments	Score range	References
Cognition (MMSE)	0–30; higher = better	[24]
Comfortable gait speed (CGS) Fast gait speed (FGS) *Time (s) to walk 15 m in comfortable and fast gait speed. For the comfortable speed walking trials, they walked at their natural speed. For the fast speed walking trials, they walked as fast as they could safely without running*	≥ 0 m/s ≥ 0 m/s	[25]
Standing balance *Foam balance test. Tested on a foam pad with eyes closed, with feet 10 cm apart and arms folded across the waist. The time until they lost balance (falling, side-stepping, hopping, pivoting, *etc*.) was measured*	0–60, second	[26]
Handgrip strength *Tested twice for the right and left hand. The best maximum contraction value of either right or left hand was used in analyses*	≥ 0 kg	[27]
Calf circumference *Measured at the point of maximum convexity of* *calf muscles*	> 0 cm	[28]
Leg/Low back pain *Self-administered question in relation to leg and low back pain, respectively: Have you been bothered by any of the following symptoms for at least three months now?*	1. No, not at all 2. Yes, a little 3. Yes, quite a lot 4. Yes, a lot	[22]
Physical activity level *Self-administered question: How much do you move and exert yourself physically during your leisure time? The question refers to the past year*	1. Hardly any physical activity 2. Mostly sitting, sometimes a walk, light gardening, or similar tasks, sometimes light household activities such as heating up food, dusting, or “clearing away 3. Light physical exercise around 2–4 h a week, such as walks, fishing, dancing, ordinary gardening including walks to and from shops. Main responsibility for light domestic work such as cooking, dusting, “clearing away,” and making beds. Performs or takes part in weekly cleaning 4. Moderate exercise 1–2 h a week, such as jogging, swimming, gymnastics, heavy gardening, home-repair, or light physical activities more than 4 h a week. Responsible for all domestic activities, light as well as heavy. Weekly cleaning with vacuum cleaning, washing floors, and window cleaning 5. Moderate exercise as least 3 h a week, such as tennis, swimming, jogging 6. Hard or very hard exercise regularly and several times a week, where the physical exertion is great, such as jogging or skiing	[23]
Smoking *Self-administered question: Are you a smoker?*	1. Yes, smoke regularly or occasionally 2. Quit, smoked 16 years or more 3. Quit, smoked 15 years or less 4. Never smoked	[23]
Alcohol consumption *Self-administered question: How often do you drink alcohol in general?*	1. Never drink alcohol 2. Once a month or less often 3. 2–4 times a month 4. 2–3 times a week 5. 4 times a week or more often	[23]

### Statistical analysis

Data were presented as means, SDs, and minimum–maximum values. The *actual* values of CGS and FGS were calculated in meters per second (m/s).

GSR was calculated as the difference between *actual* (raw) values of FGS and CGS [11]. To adjust for the differences in height of included individuals, we calculated *height-normalized* gait speeds [actual speed (m/s)/height (m)] [21]. Using these values, we calculated HN-GSR as the difference between height-normalized values of FGS and CGS. Additionally, we calculated *relative* GSR, expressed as FGS-CGS/CGS × 100% [13]. All analyses were performed for the whole study population (*n* = 4342), as well as for each of the four age groups: (I) 59–64 ys. (*n* = 2228), (II) 65–69 ys. (*n* = 691), (III) 70–79 ys. (*n* = 497) and (IV) ≥ 80 ys. (*n* = 926). The four age groups used for the analysis were created based on, and balanced by, the age-intervals and the distribution and number of subjects in each examined age subgroup (60, 66, 72, 78, 81, 84, 87, 90, and 93) from the three surveys (see the “Study population” section). We also estimated corresponding values separately for males (*n* = 2095) and females (*n* = 2247).

The study employs linear mixed models (LMMs), also known as multilevel models, to investigate the association between HN-GSR in the whole population and across various age groups (random effect part of analysis). The fixed effects represent the average impact of predictors across the entire study population, while the random effects account for variability at each age group level, and their variances provide insights into the specific extent of variability within each age group. Specifically, the model incorporates the random intercept of each age group and the random slope for both physical activity level and handgrip strength. We evaluated several configurations in our modeling approach. The decision to include a random slope for the physical activity level and handgrip strength variables as random slopes was primarily guided by two criteria: the model’s convergence, to ensure the reliability and stability of our estimates, and the Bayesian Information Criterion (BIC) to select a model that was a good fit for our data. We also provided the Marginal *R*2, which represents the variance explained by the fixed factors part of the model (i.e., the proportion of variance explained by the fixed factors alone) [29].

All numerical variables, including the dependent variable, were standardized by centering (subtracting the mean) and scaling (dividing by the standard deviation). This standardization means that our model’s interpretations are framed in terms of standard deviations, providing insights into the effects of variables relative to their variability. We also applied the Yeo-Johnson transformation to the dependent variable HN-GSR, as well as to the following numerical variables: cognition and standing balance. This transformation effectively normalizes these variables, mitigating issues related to non-normal distributions.

In many research scenarios, including ours, individuals within a specific group are likely to experience mutual influences. These influences can manifest as shared environmental factors, similar lifestyle choices, or common health trends within the group. Consequently, individuals in a specific age group are not only affected by individual-specific factors but also by the characteristics of the group to which they belong. Utilizing multilevel modeling allows us to consider these intra-group correlations and comprehend how individual factors interact with group-level influences. By incorporating random effects for different age groups, our model acknowledges and quantifies the variations within and between these groups [30, 31]. Following our choice of linear mixed models for the analysis, we confronted the challenge of missing data in our dataset. To address this, we employed the Multiple Imputation by Chained Equations (MICE) framework, a widely recognized and robust method for handling missing data in statistical analysis. We utilized elastic net regularization to identify a subset of relevant variables that contribute significantly to the model, thereby enhancing its interpretability and practical applicability. The model fit was evaluated using both the residual plots and through the Bayesian information criteria (BIC).

IBM SPSS software version 25.0 was used to perform descriptive statistical analyses, while for our statistical analysis of LMMs, we utilized the Python programming language with the statsmodels package (version 0.14.0).

The utilized Python code can be found at https://github.com/ranibasna/GaitSpeedResearve.

## Results

The mean age (SD) of all participants (*n* = 4342) was 67.8 (9.3) years and included 52% (*n* = 2247) females. The mean age (SD) of study groups I–IV was: 60.4 (0.5), 66.2 (0.4), 74.8 (3.0) and 82.9 (3.1), respectively. Age groups I and IV demonstrated the highest, respectively, the lowest mean (SD) *actual* CGS (m/s): 1.5 (0.2) and 1.2 (0.2). Corresponding values for *actual* FGS (m/s) were 1.9 (0.3) and 1.5 (0.3). *Actual* GSR (mean; SD; m/s) was highest in age groups I–II [0.4 (0.2)] and lowest in groups III–IV [0.3 (0.2)]. *Height–normalized* CGS (mean; SD; m/s) was highest in age group I [0.9 (0.1] and lowest in age group IV [0.7 (0.1)]. Corresponding values for *height–normalized* FGS (m/s) were highest in age groups I–II [1.1 (0.2)] and lowest in age groups III–IV [0.9 (0.2)]. HN-GSR (mean; SD; m/s) values were highest in age group I [0.3 (0.1)] and lowest in age groups II–IV [0.2 (0.1)]. Further details are presented in Table 2. Corresponding results, separately for males and females, are presented in the supplementary information, Tables S1 and S2.

**Table 2:** Sample characteristics for different elderly groups (years), *n* = 4342

	Whole group	Age group I 59–64 years	Age group II 65–69 years	Age group III 70–79 years	Age group IV ≥ 80 years
Number of participants	4342	2228	691	497	926
Age, years	67.8 (9.3; 59.2–94.9)	60.4 (0.5; 59.2–62.5)	66.2 (0.4; 65.3–68.7)	74.8 (3.0; 71.1–79.8)	82.9 (3.1; 80.2–94.9)
Female, *n* (%)	2247 (52)	1108 (50)	359 (52)	268 (54)	512 (55)
Cognition (MMSE, 0–30, higher = better)	27.2 (2.6; 1–30)	27.8 (2.1; 10–30)	27.4 (2.3; 14–30)	26.5 (2.8; 5–30)	26.0 (3.0; 1–30)
Comfortable gait speed (m/s)					
*Actual*	1.4 (0.3; 0.3–2.8)	1.5 (0.2; 0.6–2.8)	1.4 (0.2; 0.6–2.2)	1.3 (0.2; 0.7–2.1)	1.2 (0.2; 0.3–2.0)
*Height–normalized *^*a*^	0.8 (0.1; 0.2–1.5)	0.9 (0.1; 0.4–1.5)	0.8 (0.1; 0.4–1.3)	0.8 (0.1; 0.4–1.1)	0.7 (0.1; 0.2–1.3)
Fast gait speed (m/s)					
*Actual*	1.8 (0.4; 0.3–3.9)	1.9.(0.3; 0.7–3.9)	1.8 (0.3; 0.7–3.0)	1.6 (0.3; 0.8–3.1)	1.5 (0.3; 0.3–2.6)
*Height–normalized* ^a^	1.0 (0.2; 0.2–2.1)	1.1 (0.2; 0.5–2.1)	1.1 (0.2; 0.5–1.7)	0.9 (0.2; 0.5–1.6)	0.9 (0.2; 0.2–1.5)
Gait Speed Reserve (m/s) ^b^					
*Actual*	0.4 (0.2; 0.0–2.2)	0.4 (0.2; 0.0–2.2)	0.4 (0.2; 0.1–1.5)	0.3 (0.2; 0.0–1.3)	0.3 (0.2; 0.0–1.1)
*Height–normalized* (HN-GSR) ^a^	0.2 (0.1; 0.0–1.3)	0.3 (0.1; 0.0–1.3)	0.2 (0.1; 0.0–0.9)	0.2 (0.1; 0.0–0.7)	0.2 (0.1; 0.0–0.6)
*Relative* Gait Speed Reserve ^c^	26.8 (15.5); 0–222)	29.2 (16.5; 0–222)	25.7 (14.2; 0–108)	23.3 (12.8; 0–87)	23.7 (14.1; 0–150)
Balance pad standing (0–60, s)	49.8 (19.8; 0–60)	56.7 (11.8; 0–60)	53.7 (16.0; 0–60)	44.0 (23.0; 0–60)	32.7 (24.9; 0–60)
Handgrip strength (kg)	29.7 (12.4; 0.4–75.8)	33.2 (12.8; 0.4–75.8)	30.4 (11.5; 3.0–69.4)	26.6 (10.3; 7.0–59.3)	22.6 (9.2; 1.0–67.9)
Calf circumference (cm)	37.3 (3.3; 14.2–54.3)	37.9 (3.1; 14.2–53.0)	37.2 (3.2; 29–54.3)	36.6 (3.2; 25.9–52.2)	36.6 (3.1; 24.5–49.5)
Weight (kg)	76.8 (15.0; 35.0–146.6)	78.9 (15.7; 39.0–146.6)	78.1 (14.4; 41.0–139.9)	75.2 (13.4; 35.0–122.2)	71.4 (12.9; 36.8–117.0)
Leg pain severity (1–4, higher = more)	1 (1–2; 1–4)	1 (1–2; 1–4)	1 (1–2; 1–4)	2 (1–2; 1–4)	2 (1–2; 1–4)
Back pain severity (1–4, higher = more)	2 (1–2; 1–4)	1 (1–2; 1–4)	2 (1–2; 1–4)	2 (1–2; 1–4)	2 (1–2; 1–4)
Physical activity level (1–6, higher = better)	3 (3–4; 1–6)	3 (3–4; 1–6)	3 (3–4; 1–6)	3 (3–4; 1–6)	3 (3–4; 1–6)
Smoking (1–4, higher = less)	3 (2–4; 1–4)	3 (2–4; 1–4)	3 (2–4; 1–4)	3 (2–4; 1–4)	4 (2–4; 1–4)
Alcohol consumption (1–5, higher = more)	3 (2–4; 1–5)	3 (2–4; 1–5)	3 (2–4; 1–5)	2 (1–3; 1–5)	2 (1–3; 1–5)

Data presented as means (standard deviation) or median(q1-q3) and minimum–maximum values

^**a**^*Height–normalized* gait speed: actual gait speed (m/s)/height (m)

^b^Gait Speed Reserve: difference between fast gait speed (FGS) and comfortable gait speed (CGS)

^c^*Relative* Gait Speed Reserve: [FGS–CGS/CGS] × 100

*kg*, kilogram; *MMSE*, Mini Mental State Examination; *m/s*, meters per second; *s*, second

The following independent variables were associated with HN-GSR in the fixed part of LMMs analysis: gender, cognition, standing balance, handgrip strength, calf circumference, weight, leg pain severity, physical activity level, tobacco and alcohol consumption. The strongest positive association with HN-GSR was observed for handgrip strength (B = 0.249, 95%CI: 0.198–0.301), which means that each standard deviation increases in handgrip strength is linked to a 0.249 standard deviation increase in Yeo-Johnson transformation of HN-GSR. The other factors linked to an increase in HN-GSR (stated according to the strength of the contribution) were: less smoking, better cognition, better standing balance, higher alcohol consumption, higher levels of physical activity and larger calf circumference. The strongest negative associations with HN-GSR were observed for female gender (B =  − 0.228; 95%CI: − 0.310 to − 0.147), followed by higher weight and more leg pain. The Marginal R2 (the proportion of variance explained by the fixed factors alone) implies that this model explained 26% of the variance in HN-GSR. The Random part of LMMs analyses showed that physical activity level and handgrip strength had different effects on HN-GSR across age groups (I–IV). The random effect coefficients for each of these associated factors (physical activity and handgrip strength) were 0.001 (Table 3). Values for the random slopes (coefficients) (Table 4) show the magnitude of deviation from fixed effects values (Table 3). For example, the random slope for physical activity levels in age groups I and II is 0.003, slightly enhancing the fixed effect, while in age groups III and IV, the random slope is − 0.003, diminishing the fixed effect. In general, the random slopes indicate only minor deviations from the average effect across age groups.

**Table 3:** Results from linear mixed models (LMMs) with height-normalized gait speed reserve (HN-GSR) as a dependent variable, *n* = 4342

Factors	Standardized B (95%CI)	*P*-value
*Fixed effect*		
Age group (I–IV)	− 0.097 (− 0.394, 0.200)	0.523
Female gender	− 0.228 (− 0.310, − 0.147)	< 0.001
Cognition (higher = better)	0.124 (0.096, 0.151)	< 0.001
Balance pad standing (0–60, s)	0.118 (0.089, 0.148)	< 0.001
Handgrip strength (kg)	0.249 (0.198, 0.301)	0.003
Calf circumference (cm)	0.049 (0.012, 0.086)	0.001
Weight (kg)	− 0.173 (− 0.215, − 0.132)	< 0.001
Leg pain severity (higher = more)	− 0.112 (− 0.149, − 0.075)	< 0.001
Back pain severity (higher = more)	0.030 (− 0.065, 0.004)	0.086
Physical activity level (higher = better)	0.052 (0.006, 0.099)	0.027
Smoking		
Smoke regularly or occasionally	reference	
Quit, smoked 16 years or more	0.127 (0.027, 0.187)	0.009
Quit, smoked 15 years or less	0.141 (0.054, 0.228)	0.006
Never smoked	0.155 (0.079–0.231)	< 0.001
Alcohol consumption (higher = more)	0.058 (0.035, 0.082)	< 0.001
*Random effect*		
Age group variance	0.137	
Covariance of age group and physical activity level	− 0.003	
Physical activity level variance	0.001	
Covariance of age group and handgrip strength	0.003	
Covariance of physical activity level and handgrip strength	0.000	
Handgrip strength variance	0.001	

Marginal *R*^2^ = 0.26 for fixed effect

**Table 4:** Results from random part of linear mixed models (LMMs) with height-normalized gait speed reserve (HN-GSR) as a dependent variable in relation to age groups, *n* = 4342

	Random slopes (coefficients)	
	Physical activity level	Handgrip strength
Age group I 59–64 years	0.003	0.011
Age group II 65–69 years	0.003	− 0.005
Age group III 70–79 years	− 0.003	− 0.013
Age group IV ≥ 80 years	− 0.003	0.007

## Discussion

By evaluating four age groups in an elderly population-based cohort, we found gender and nine modifiable factors as significantly associated with HN-GSR. Handgrip strength, cognition, standing balance, physical activity, calf circumference, and alcohol habits had positive associations with HN-GSR while age, tobacco use, female gender, leg pain, and weight showed the opposite.

Physical activity and handgrip strength varied across age groups in their impact on HN-GSR. The differences were, however, comparatively minor.

*Actual and height-normalized* values of gait speeds observed in our study were largely in line with previous studies [4, 5, 9, 21, 32–34]. GSR for the older population was previously evaluated in some studies [9, 11–13] and varied between 0.6 and 0.2 m/s. Diversity in reported results may be related to differences in gait speed assessment methods, inclusion criteria, and anthropometrics [33]. However, when compared with corresponding subgroups in our study population, the results are largely compatible. To the best of our knowledge, mean HN-GSR values were not reported before. However, *height-normalized* FGS and CGS tend to decrease after adjusting for height, so our mean HN-GSR of 0.2 m/s is reasonable.

Our findings regarding associations between female gender and decreased HN-GSR are in line with previous findings for GSR [35]. Females’ poorer ability to perform beyond the basic level and walk faster may depend on well-known fundamental biological mechanisms contributing to sex differences that are also reflected in more pronounced frailty among women [36]. Higher handgrip strength and better cognition, both strongly associated with increased HN-GSR, were previously reported as significant for increased GSR [9]. Indeed, sarcopenia of which handgrip strength is an indicative measure [37, 38], has been associated with reduced gait speed outcomes in older people [39, 40]. Handgrip strength was also suggested as a viable substitute for many functional tests involving the lower limb that are circular for assessment of balance and gait in older population [41]. In addition, the movement speed of the upper limbs was a significant determinant of FGS, suggesting that the ability to move any region rapidly might be a critical factor in FGS speed. This is related to one’s general ability to generate muscle power and perform an activity quickly and repeatedly by applying a given force [42].

The potential explanation for the significant association between cognition and HN-GSR might be that the ability to walk faster needs greater cognitive resources to maintain balance and rapidly take in changing surroundings [43]. This is also in line with Callisaya et al. findings who showed, by evaluating subgroups with different cognitive states, that poorer cognition was associated with decreased GSR [13]. This confirms the previously suggested hypothesis that GSR, as well as gait speeds, are multisystem phenomena [9, 44]. Associations between GSR and cognition also support the cognitive reserve concept [45]. Significant associations were reported between physical frailty, or gait speed, and cognitive frailty. Thus, the ability to perform beyond the basal physical level may be dependent on cognitive resources beyond the basal level. This suggestion is in line with previously reported positive associations of educational level with GSR [9].

We also reported some novel findings. Calf circumference, a well-known indicator of loss of muscle mass due to sarcopenia is also related to ankle plantar flexor strength in healthy older population [46]. The ability to maintain balance during a single support phase of gait, pushing of and moving forward, requires adequate strength of various lower limb muscles, including plantar flexion generated from calf muscles [47, 48]. This force, also called ankle power, is suggested to be the primary cause of age-related reduction in gait speed [49–51]. Importantly, impaired ankle power was significantly associated with decreased GSR in individuals after traumatic brain injury that may have prominent push-of phase deficit [52]. The association between calf circumference and increased HN-GSR was, however, relatively weak in our study. That might be explained by a combination of opposite effects. Partly, as an indicator of sarcopenia in the very oldest, which affects gait speeds, and generally as an indicator of poor physical performance, especially among women [46]. On the other hand, in the legs of overweight subjects’ muscular fat infiltration could increase calf circumference but not HN-GSR [53]. In fact, higher weight in our study population indicated decreased HN-GSR. This result is in line with previous findings suggesting negative associations between Body Mass Index (BMI) and GSR [35]. That is in line with previous studies in elderly disputing suggestions that being overweight in older age is protective of mobility [54].

Another novel finding was the contribution of better standing balance to increased HN-GSR. Standing balance was investigated by standing on a foam surface with eyes closed [55]. This test implies that both proprioceptive and visual input are limited, and the subject must rely mostly on their vestibular function. The foam test is suggested to be a suitable method of screening adults for vestibular disorders [56]. Progressive loss of hair cells, fragmentation of otoconia, reduction of numbers of cells in the vestibular nuclei, and loss of afferent fibers are natural processes of normal aging that begin already in middle age [57]. The vestibular system plays a critical role in the modulation of gait [58]. Previous research indicates that the otolith organs contribute to acceleration cues to control different modalities of gait speeds [59], which may partly explain our findings. However, other age-related conditions may contribute to impaired standing balance. Early signs of peripheral neuropathy, a common but poorly recognized condition among older adults, were also associated with a poor ability to manage the foam test. That may be related to an early sensory loss in the foot and legs and limited proprioceptive input [26, 60]. Also, conditions affecting the extrapyramidal system may aggravate standing balance due to impaired sensorimotor integration [61]. Particularly standing on an unstable surface with eyes closed is challenging, even though the vestibular system is normal [62]. Importantly, mild parkinsonian signs and more advanced parkinsonism were highly frequent in a general population of older adults [63].

Chronic back and leg pain are common problems in community-dwelling older adults which was reflected in our results. Associations between pain, particularly in multiple locations, such as back, hip, knee and foot, and decreased gait speed were reported before [64]. Our results showed that median back pain severity was higher than leg pain severity. However, the severity of leg pain, not back pain, was associated with decreased HN-GSR. The contribution of leg pain may be related to several conditions, such as referred pain from the lower back or arthritis [65, 66]. Foot pain is also common in older adults. A meta-analysis of 31 studies (including 75,505 participants) estimated a foot pain prevalence to 24% [67]. Additionally, early signs of peripheral neuropathy may be associated with pain, burning, or aching in legs or feet, or prickling sensations in legs, or feet [26, 60]. Other painful conditions are foot deformities (hallux valgus, claw, hammer or overlapping toes), skin and nail pathologies, infections, and circulatory problems [68, 69]. Painful foot deformities are highly represented in subjects with extrapyramidal conditions, which were reported in more than 80% of the very oldest [63, 70].

Lifestyle, including physical activity level, smoking habits, and alcohol consumption is associated with physical and cognitive functioning [20]. In line with this, higher levels of physical activity and less smoking were associated with increased HN-GSR. However, we observed a paradoxical positive association between higher alcohol consumption and HN-GSR. In population-based studies, based on voluntary participation and on invitation, the number of heavy drinkers is underrepresented. The higher alcohol consumption in our population might represent moderate drinkers. The trend of poorer gait performance in non-drinkers has been observed previously, probably confounded by comorbidity [71, 72]. A longitudinal study design should probably be of value to examine the effects of alcohol consumption on HN-GSR over time.

The differences across age groups imply that younger participants, aged 59–69 years, had slightly higher associations between physical activity and HN-GSR, compared to older participants. The lesser effects of physical activity in older groups may be due to an age-related cognitive decline that may compromise the ability to walk fast [43]. The effect of handgrip strength across age groups was highest in the youngest and the oldest groups which may reflect different group-related conditions; in the youngest the fitness level and in the oldest the sarcopenia spectrum. The effects of physical activity and handgrip strength across age groups were, however, comparatively minor.

To sum up, associated factors with HN-GSR are largely in line with those previously reported for GSR [9, 35]. From a generalizability point of view, the results regarding handgrip strength need attention because of its significance for HN-GSR and GSR in large study populations, our and Davis et al. [9], respectively.

HN-GSR/GSR are complex constructs that may reflect the capacity to go beyond the comfort zone but does not necessarily rule out the frailty. Indeed, the extraordinary difference between FGS and CGS may be related to unhealthy risk-taking. The mean *relative* GSR in our study was about 27%, but in all age groups, there occurred participants with very high values, as high as 200%. Such high values were suggested to occur in individuals with greater cognitive impairment [13]. Further work is, however, needed to find out if measuring HN-GSR/GSR may be useful in identifying adverse health outcomes and if it is informative on overall physiological reserve.

### Clinical implications

Measuring HN-GSR/GSR has several clinical implications because it probably reflects a complex interaction between several physiologic impairments before each one could express itself as a complete clinical manifestation [12, 73]. Our results imply that attention should be directed to calf circumference/ankle power, standing balance, leg and feet pain, weight, as well as tobacco and alcohol consumption. HN-GSR/GSR in women requires particular attention due to significant associations with gender-related decline. Associations with handgrip strength and cognition imply that HN-GSR/GSR may also act as an indicator of overall diminished physiological reserve and frailty [74]. Importantly, identified associated factors are modifiable through engaging in physical exercise, cognitive stimulation, and lifestyle changes. HN-GSR/GSR may also have important safety implications during daily life activities such as running for the bus or crossing the road [13].

### Strength and limitations

A major strength of our study is its large population with both rural and urban populations sampled. Aid was also offered to participants who had difficulties answering the questionnaires due to language difficulties, visual impairment, or other disabilities. Participants who could not visit the research clinics were offered a home visit; however, gait speed could not be measured in these subjects, nor in those visited in nursing homes. Therefore, healthy, independent, and overall, cognitively intact participants are over-represented in the study samples. It is also important to be aware of possible cohort effects in older men and women when assessing the results of physical tests, but its influence is unclear. On the one side, the proportion of those who did not participate in the GÅS study (the most fragile) was higher in the earlier-born cohort [75]. On the other side, it has previously been observed, when examining a smaller group representing 60- and 81-year-old participants in two cohorts born twelve years apart, that the later-born cohort performed moderately better than the earlier-born in most of the physical tests [75, 76]. The exception in both age groups/substudies was handgrip strength, which showed no significant differences between the birth cohorts. However, the suggested cohort effect might be less significant in our study when using all subjects from three GÅS-study’s examination waves, because each wave took 3.5 years to perform and was followed immediately by the next wave, which means that the birthdate difference of subjects at the same age examined in the same wave was as wide as between the adjacent waves.

## Conclusions

Associated factors with HN-GSR represented multi-domain features that are in line with previous findings reported for GSR. Further work is needed to find out if measuring HN-GSR/GSR may be useful in predicting adverse health outcomes and if it is informative on overall physiological reserve. Measuring HN-GSR/GSR may help clinicians to early identify physiological impairments such as strength or balance deficits, impaired cognition, pain, or unhealthy lifestyle habits, especially among women. HN-GSR/GSR may also have safety implications in daily life activities.

## Supplementary Information

Below is the link to the electronic supplementary material.

Supplementary file1 (DOCX 27 KB)

## Data Availability

The authors confirm that the data supporting the findings of this study are available in the article.
