# Supplementary material for: Gait Speed Reserve in the general population-based ‘Good Aging in Skåne’ cohort study—distribution and associated factors
Source: GeroScience. 2024 Aug 27;47(1):965–76. doi: 10.1007/s11357-024-01318-6 (PMC11872813; doi:10.1007/s11357-024-01318-6)
Supplement: Supplementary file 1 — Supplementary file1 (DOCX 27 KB) [file 11357_2024_1318_MOESM1_ESM.docx]

**Table S1. Sample characteristics for males in different older groups (years), n=2095**

|  | All Males | Age group I  59–64 years | Age group II  65–69 years | Age group III  70–79 years | Age group IV  ≥80 years |
| --- | --- | --- | --- | --- | --- |
| Number of participants | 2095 | 1120 | 332 | 229 | 414 |
| Age, years | 67.3 (9.1; 59.1–93.1) | 60.4 (0.5; 59.1–62.4) | 66.2 (0.4; 65.3–68.7) | 74.9 (3.1; 71.3–79.0) | 82.9 (3.1; 80.1–93.1) |
| Cognition (MMSE, 0–30, higher=better) | 27.2 (2.4; 8–30) | 27.8 (2.0; 10–30) | 27.4 (2.3; 14–30) | 26.5 (2.7; 13–30) | 26.1 (2.9; 8–30) |
| Comfortable gait speed (m/s) |  |  |  |  |  |
| *Actual* | 1.4 (0.2; 0.6–2.8) | 1.5 (0.2; 0.8–2.8) | 1.5 (0.2; 0.8–2.2) | 1.3 (0.2; 0.7–2.1) | 1.2 (0.2; 0.6–2.0) |
| *Height–normalized* ^a^ | 0.8 (0.1; 0.4–1.5) | 0.9 (0.1; 0.4–1.5) | 0.8 (0.1; 0.5–1.3) | 0.7 (0.1; 0.4–1.1) | 0.7 (0.1; 0.4–1.1) |
| Fast gait speed (m/s) |  |  |  |  |  |
| *Actual* | 1.9 (0.4; 0.7–3.9) | 2.0 (0.3; 0.9–3.9) | 1.9 (0.3; 1.0–3.0) | 1.7 (0.3; 0.9–3.1) | 1.6 (0.3; 0.7–2.6) |
| *Height–normalized* ^a^ | 1.1 (0.2; 0.4–2.0) | 1.1 (0.2; 0.5–2.0) | 1.1 (0.2; 0.6–1.7) | 0.9 (0.2; 0.5–1.6) | 0.9 (0.2; 0.4–1.5) |
| Gait Speed Reserve (m/s) ^b^ |  |  |  |  |  |
| *Actual* | 0.4 (0.2; 0.0–2.2) | 0.5 (0.3; 0.0–2.2) | 0.4 (0.2; 0.1–1.5) | 0.3 (0.2; 0.0–1.3) | 0.3 (0.2; 0.0–1.1) |
| *Height–normalized* (HN-GSR) ^a^ | 0.2 (0.1; 0.0–1.3) | 0.3 (0.1; 0.0–1.3) | 0.2 (0.1; 0.0–0.9) | 0.2 (0.1; 0.0–0.7) | 0.2 (0.1; 0.0–0.6) |
| *Relative* Gait Speed Reserve ^c^ | 31.1 (17.2; 0–222) | 33.8 (18.7; 0–222) | 29.9 (15.0; 3–108) | 26.5 (14.2; 2–87) | 27.3 (14.4; 0–121) |
| Balance pad standing (0–60, s) | 51.8 (18.3; 0–60) | 57.6 (10.4; 0–60) | 54.4 (15.3; 0–60) | 47.2 (21.6; 0.0–60) | 36.3 (25.0; 0–60) |
| Handgrip strength (kg) | 38.8 (10.4; 0.4–75.8) | 42.8 (9.8; 0.4–75.8) | 39.6 (8.7; 13.2–69.4) | 34.7 (8.4; 11.5–59.3) | 29.7 (7.3; 10.3–44.7) |
| Calf circumference (cm) | 37.9 (3.0; 24.5–53.0) | 38.5 (2.9; 30.0–53.0) | 37.8 (3.0; 29.0–47.2) | 37.1 (2.8; 29.0–51.2) | 36.6 (2.9; 24.5–49.5) |
| Weight (kg) | 84.3 (13.5; 47.0–146.6) | 86.6 (14.1; 45.0–146.6) | 85.0 (13.2; 55.7–139.9) | 82.4 (11.9; 55.2–122.2) | 78.7 (11.3; 49.6 –117.0) |
| Leg pain severity (1–4, higher=more) | 1 (1–2; 1–4) | 1 (1–2; 1–4) | 1 (1–2; 1–4) | 1 (1–2; 1–4) | 3 (2–3; 1–4) |
| Back pain severity (1–4, higher=more) | 1 (1–2; 1–4) | 1 (1–2; 1–4) | 1 (1–2; 1–4) | 1 (1–2; 1–4) | 1 (1–2; 1–4) |
| Physical activity level (1–6, higher=better) | 3 (3–4; 1–6) | 3 (3–4; 1–6) | 3 (3–4; 1–6) | 3 (3–4; 1–6) | 3 (3–4; 1–6) |
| Smoking (1–4, higher=less) | 2 (2–4; 1–4) | 2 (2–4; 1–4) | 2 (2–4; 1–4) | 2 (2–4; 1–4) | 2 (2–4; 1–4) |
| Alcohol consumption (1–5, higher=more) | 3 (2–4; 1–5) | 3 (2–4; 1–5) | 3 (2–4; 1–5) | 3 (2–4; 1–5) | 2 (2–3; 1–5) |

Data presented as means (standard deviation) or median(q1-q3) and minimum–maximum values.

**^a^**Height–normalized gait speed: actual gait speed (m/s)/height (m).

^b^Gait Speed Reserve: difference between fast gait speed (FGS) and comfortable gait speed (CGS).

^c^*Relative* Gait Speed Reserve [FGS–CGS/CGS] x 100.

Kg, kilogram; MMSE, Mini Mental State Examination; m/s, meters per second; s, second.

**Table S2. Sample characteristics for females in different older groups (years), n=2247**

|  | All females | Age group I  59–64 years | Age group II  65–69 years | Age group III  70–79 years | Age group IV  ≥80 years |
| --- | --- | --- | --- | --- | --- |
| Number of participants | 2247 | 1108 | 359 | 268 | 512 |
| Age, years | 68.2 (9.4; 59.2–94.0) | 60.4 (0.5; 59.2–62.5) | 66.2 (0.4; 65.5–67.8) | 74.7 (3.0; 71.1–79.8) | 83.0 (3.1; 80.2–94.9) |
| Cognition (MMSE, 0–30, higher=better) | 27.2 (2.6; 5–30) | 27.8 (2.3; 13–30) | 27.4 (2.3; 15–30) | 26.6 (2.8; 5–30) | 26.0 (3.2; 1–30) |
| Comfortable gait speed (m/s) |  |  |  |  |  |
| *Actual* | 1.4 (0.3; 0.6–2.4) | 1.5 (0.2; 0.6–2.4) | 1.4 (0.2; 0.6–2.1) | 1.2 (0.2; 0.7–1.8) | 1.1 (0.2; 0.3–2.0) |
| *Height–normalized* ^a^ | 0.8 (0.15; 0.4–1.5) | 0.9 (0.1; 0.4–1.5) | 0.9 (0.1; 0.4–1.2) | 0.8 (0.1; 0.4–1.1) | 0.7 (0.1; 0.2–1.3) |
| Fast gait speed (m/s) |  |  |  |  |  |
| *Actual* | 1.7 (0.3; 0.7–3.4) | 1.8 (0.3; 0.7–3.4) | 1.7 (0.3; 0.7–2.4) | 1.5 (0.2; 0.8–2.5) | 1.4 (0.3; 0.3–2.4) |
| *Height–normalized* ^a^ | 1.0 (0.2; 0.5–2.1) | 1.1 (0.2; 0.5–2.1) | 1.0 (0.2; 0.5–1.5) | 0.9 (0.1; 0.5–1.5) | 0.9 (0.2; 0.2–1.5) |
| Gait Speed Reserve (m/s) ^b^ |  |  |  |  |  |
| *Actual* | 0.3 (0.16; 0.0–1.2) | 0.4 (0.2; 0.0–1.1) | 0.3 (0.2; 0.0–1.2) | 0.3 (0.1; 0.0–0.7) | 0.2 (0.1; 0.0–0.1) |
| *Height–normalized* (HN-GSR) ^a^ | 0.2 (0.1; 0.0–0.7) | 0.2 (0.1; 0.0–0.6) | 0.2 (0.1; 0.0–0.7) | 0.2 (0.1; 00–0.4) | 0.1 (0.1; 0.0–0.6) |
| *Relative* Gait Speed Reserve ^c^ | 22.8 (12.3; 0–150) | 24.6 (12.2; 0–130) | 21.9 (12.3: 0–103) | 20.5 (10.8; 0–70) | 20.8 (13.1; 0–150) |
| Balance pad standing (0–60, s) | 48.0 (21.0; 0–60) | 55.9 (13.0; 0–60) | 53.1 (16.7; 0–60) | 41.2 (23.8; 0–60) | 29.8 (24.5; 0–60) |
| Handgrip strength (kg) | 21.2 (6.6; 3.0–67.9) | 23.4 (6.5; 3.0–44.9) | 21.8 (5.7; 3.0–35.8) | 19.6 (5.4; 7.0–33.0) | 16.8 (5.9; 1.0–67.9) |
| Calf circumference (cm) | 36.7 (3.4; 14.2–54.3) | 37.4 (3.3; 14.2–53.0) | 36.8 (3.4; 29.0–54.3) | 36.2 (3.4; 25.9–52.9) | 35.6 (3.2; 27.5–49.5) |
| Weight (kg) | 69.8 (12.7; 35.0–132) | 71.3 (13.4; 39.0–132.0) | 71.7 (12.5; 41.0–113.7) | 68.9 (11.4; 35.0–116.0) | 65.5 (10.8; 36.8–110.0) |
| Leg pain severity (1–4, higher=more) | 2 (1–2; 1–4) | 1 (1–2; 1–4) | 1 (1–2; 1–4) | 2 (1–2; 1–4) | 2 (1–2; 1–4) |
| Back pain severity (1–4, higher=more) | 2 (1–2; 1–4) | 2 (1–2; 1–4) | 2 (1–2; 1–4) | 2 (1–2; 1–4) | 2 (1–2; 1–4) |
| Physical activity level (1–6, higher=better) | 3 (3–4; 1–6) | 3 (3–4; 1–6) | 3 (3–4; 1–6) | 3 (3–4; 1–6) | 3 (3–3; 1–5) |
| Smoking (1–4, higher=less) | 3 (2–4; 1–4) | 3 (2–4; 1–4) | 3 (2–4; 1–4) | 4 (2–4; 1–4) | 4 (3–4; 1–4) |
| Alcohol consumption (1–5, higher=more) | 2 (2–3; 1–5) | 3 (2–4; 1–5) | 3 (2–3; 1–5) | 2 (1–3; 1–5) | 2 (1–3; 1–5) |

Data presented as means (standard deviation) or median(q1-q3) and minimum–maximum values.

**^a^**Height–normalized gait speed: actual gait speed (m/s)/height (m).

^b^Gait Speed Reserve: difference between fast gait speed (FGS) and comfortable gait speed (CGS).

^c^*Relative* Gait Speed Reserve: [FGS–CGS/CGS] x 100.

Kg, kilogram; MMSE, Mini Mental State Examination; m/s, meters per second; s, second.
